# Randomized Comparison of Two New Methods for Chest Compressions during CPR in Microgravity—A Manikin Study

**DOI:** 10.3390/jcm11030646

**Published:** 2022-01-27

**Authors:** Jan Schmitz, Anton Ahlbäck, James DuCanto, Steffen Kerkhoff, Matthieu Komorowski, Vanessa Löw, Thais Russomano, Clement Starck, Seamus Thierry, Tobias Warnecke, Jochen Hinkelbein

**Affiliations:** 1 Department of Anesthesiology and Intensive Care Medicine, University Hospital of Cologne, 50937 Cologne, Germany; steffen.kerkhoff@uk-koeln.de (S.K.); Vanessa.loew1@uk-koeln.de (V.L.); jochen.hinkelbein@uk-koeln.de (J.H.); 2 Working Group Emergency Medicine and Air Rescue, German Society of Aviation and Space Medicine (DGLRM), 80331 Munich, Germany; 3 Working Group Standards, Recommendations, and Guidelines, German Society of Aviation and Space Medicine (DGLRM), 80331 Munich, Germany; 4 Space Medicine Group, European Society of Aerospace Medicine (ESAM), 50937 Cologne, Germany; clementstarck@gmail.com (C.S.); seam.thi@gmail.com (S.T.); 5 Department of Sleep and Human Factors Research, Institute of Aerospace Medicine, German Aerospace Center, 51149 Cologne, Germany; 6 Department of Anesthesia and Intensive Care, Örebro University Hospital, 701 85 Örebro, Sweden; anton.ahlback@gmail.com; 7 Department of Anesthesiology, Aurora St. Luke’s Medical Center, Milwaukee, WI 53215, USA; jducanto@mac.com; 8 Department of Surgery and Cancer, Faculty of Medicine, Imperial College London, London SW7 2AZ, UK; matthieu.komorowski@gmail.com; 9 Centre for Human and Applied Physiological Sciences, School of Basic and Medical Biosciences, Faculty of Life Sciences & Medicine, King’s College London, London SE1 9RT, UK; trussomano@hotmail.com; 10 Anesthesiology and Intensive Care Department, University Hospital of Brest, 29609 Brest, France; 11 Anesthesiology Department, South Brittany General Hospital, 56322 Lorient, France; 12 Department of Anaesthesiology, Critical Care, Emergency Medicine and Pain Therapy, Klinikum Oldenburg, Medical Campus, University of Oldenburg, 26133 Oldenburg, Germany; tobiaswarnecke@web.de

**Keywords:** CPR, microgravity, submerged model, spaceflight, resuscitation

## Abstract

Background: Although there have been no reported cardiac arrests in space to date, the risk of severe medical events occurring during long-duration spaceflights is a major concern. These critical events can endanger both the crew as well as the mission and include cardiac arrest, which would require cardiopulmonary resuscitation (CPR). Thus far, five methods to perform CPR in microgravity have been proposed. However, each method seems insufficient to some extent and not applicable at all locations in a spacecraft. The aim of the present study is to describe and gather data for two new CPR methods in microgravity. Materials and Methods: A randomized, controlled trial (RCT) compared two new methods for CPR in a free-floating underwater setting. Paramedics performed chest compressions on a manikin (Ambu Man, Ambu, Germany) using two new methods for a free-floating position in a parallel-group design. The first method (Schmitz–Hinkelbein method) is similar to conventional CPR on earth, with the patient in a supine position lying on the operator’s knees for stabilization. The second method (Cologne method) is similar to the first, but chest compressions are conducted with one elbow while the other hand stabilizes the head. The main outcome parameters included the total number of chest compressions (*n*) during 1 min of CPR (compression rate), the rate of correct chest compressions (%), and no-flow time (s). The study was registered on clinicaltrials.gov (NCT04354883). Results: Fifteen volunteers (age 31.0 ± 8.8 years, height 180.3 ± 7.5 cm, and weight 84.1 ± 13.2 kg) participated in this study. Compared to the Cologne method, the Schmitz–Hinkelbein method showed superiority in compression rates (100.5 ± 14.4 compressions/min), correct compression depth (65 ± 23%), and overall high rates of correct thoracic release after compression (66% high, 20% moderate, and 13% low). The Cologne method showed correct depth rates (28 ± 27%) but was associated with a lower mean compression rate (73.9 ± 25.5/min) and with lower rates of correct thoracic release (20% high, 7% moderate, and 73% low). Conclusions: Both methods are feasible without any equipment and could enable immediate CPR during cardiac arrest in microgravity, even in a single-helper scenario. The Schmitz–Hinkelbein method appears superior and could allow the delivery of high-quality CPR immediately after cardiac arrest with sufficient quality.

## 1. Introduction

Space exploration and discovery will take humans far beyond low-Earth orbit (LEO). The National Aeronautics and Space Administration (NASA) and the European Space Agency (ESA) are preparing to send astronauts to the Moon (Artemis mission) to help prepare humanity for its next step—sending astronauts to Mars [1]. The journey will take up to 9 months each way causing extreme isolation and, therefore, resulting in total crew autonomy for almost 3 years [2,3].

During both Moon and Mars missions, there will be no possibility for crews to rapidly return to the ground in cases of an emergency; real-time assistance from Earth will be limited or impossible due to communication delays [4,5]. Given the delay of data transmission (up to 22 min per direction for the case of Mars), evacuation and telemedical support will not be possible/available in cases of a severe medical emergency [4,6]. 

During the journey in microgravity, the human body is subject to altered physiological conditions that are certain to significantly impact the astronaut’s health [7]. Hemodynamic maladaptation resulting in hypotension, tachycardia, or even cardiac arrythmia with the risk of severe cardiovascular disease seems to be prevalent after exposure to microgravity [8,9]. Recent data also show that structural changes occur in the brain are associated with a decline in cognitive function resulting in spaceflight-associated neuro-optic syndrome [10,11]. Moreover, in the microgravity environment, the risk of trauma-associated injuries is significant [4,12]. Estimations from analogue populations suggest that one major medical event could occur for every 900-day mission [13]. Although no cardiac arrests have been reported to date, the theoretical risk of a dangerous cardiac or neurological event occurring in microgravity remains, even if it is low due to stringent screening and extensive training of astronauts. The risk of acute and life-threatening conditions also increases with mission duration and remoteness from Earth [4].

The European Resuscitation Council (ERC) basic life support guidelines highlight that cardiac arrest without immediate compensation with chest compressions will result in irreversible cerebral damage [14]. Current guidelines recommend a compression rate of 100–120/min with a compression depth of a minimum of 50 to a maximum 60 mm. Moreover, recently published guidelines on CPR in microgravity follow mainly ERC recommendations for CPR [15]. In recent years, research has been undertaken to develop methods of CPR in microgravity; thus far, five different methods have been described [2,6,16]. 

Regarding CPR quality, the Handstand method seems to be the most effective with respect to treating cardiac arrest, but with the major limitation that it needs a diameter between the operator and the compartment [2,6,16]. If the Handstand method is unapplicable in some scenarios, the Evetts–Russomano method is an acceptable alternative because CPR quality appears to be only slightly lower [16]. However, regardless of the method used, CPR quality in (simulated) microgravity is worse in comparison to ground-based CPR. Therefore, there is a need to develop new CPR methods.

The aim of the present study is to describe two new methods for CPR in microgravity and to analyze and compare the quality of CPR achieved.

## 2. Materials and Methods

**Figure 1:**
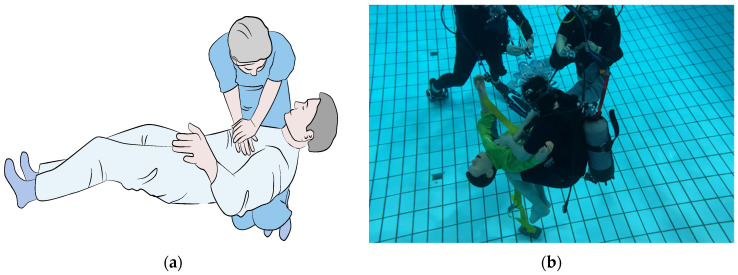
Graphic example (**a**) and execution in our submerged setting (**b**) of the Schmitz–Hinkelbein method (Graphic: Medizinfoto Köln, Photo: Jan Schmitz).

**Figure 2:**
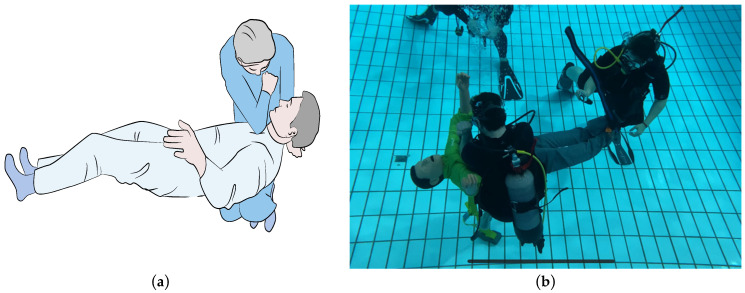
Graphic example (**a**) and execution in our submerged setting (**b**) of the Cologne method (Graphic: Medizinfoto Köln, Photo: Jan Schmitz).

We conducted a randomized parallel group trial (RCT) comparing two new methods for CPR in a free-floating underwater setting. Both methods require the operator to stabilize the patient on his/her thighs and deliver chest compressions using both arms in the first method (Schmitz–Hinkelbein method, SHM, Figure 1), or using one elbow in the Cologne method (CM, Figure 2).

### 2.1. Subjects

**Figure 3:**
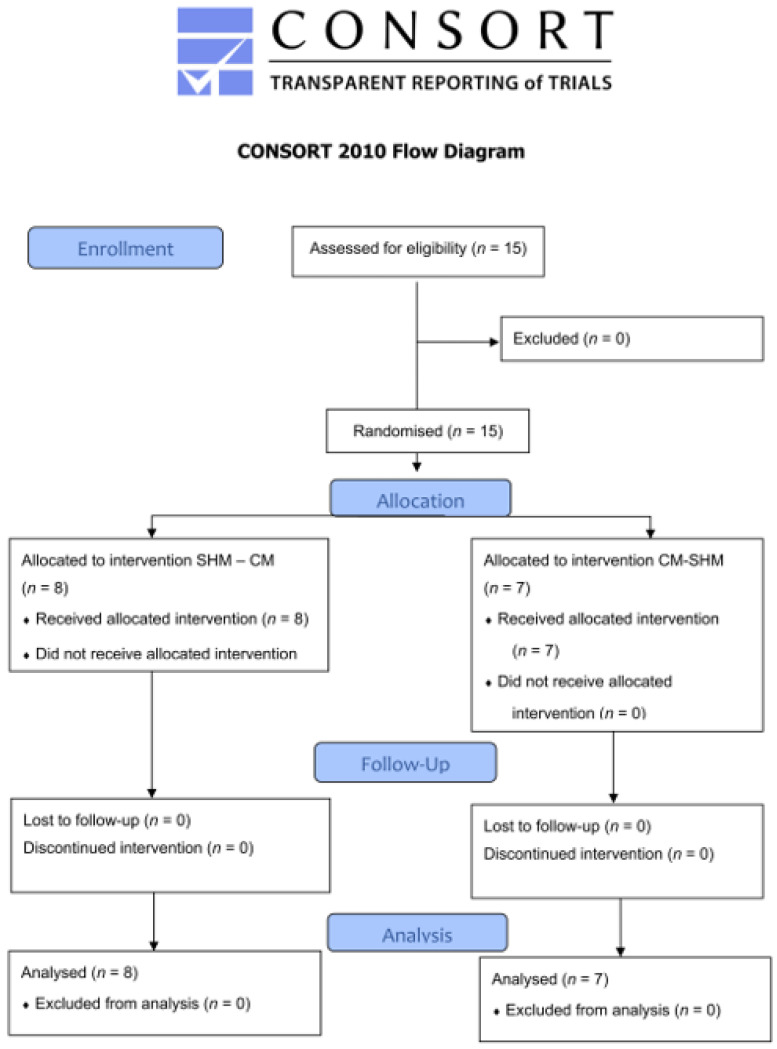
CONSORT flow diagram. SHM: Schmitz–Hinkelbein method; CM: Cologne method.

The participants were trained paramedics holding a valid diving certificate. The criteria for inclusion were EMT with valid diving certificates (SSI—Open Water Diver (OWD); CMAS *; PADI Open Water Diver; ISO 24801-2 (Autonomous Diver); or NAUI Scuba Diver) or equivalent licenses. The criteria for exclusion were any acute or chronic ear, nose, or throat disease. Figure 3 shows the enrollment process.

Written informed consent was obtained from all subjects before completing a short questionnaire to gather information about the participant’s level of experience as an EMT and with CPR, as well as their total number of dives. Moreover, current health status was checked for acute or chronic ENT diseases.

### 2.2. Setting

**Figure 4:**
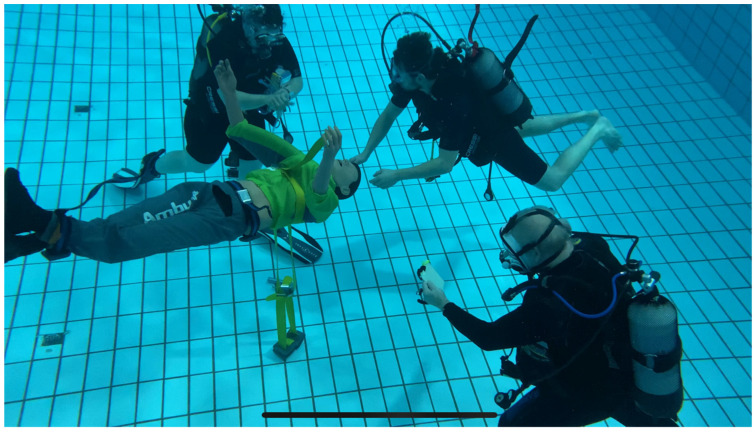
Submerged setting with manikin in free-floating position (Photo: J. Schmitz).

All participants tested both CPR methods during a single dive. Chest compressions were performed using a full-body manikin (AmbuMan^®^ Airway Wireless, Ambu Ltd., Bad Nauheim, Germany). The manikin was submerged and counterbalanced in a free-floating position approximately 1.5 m above the bottom of the pool (Figure 4). One dive instructor as well as one additional diver accompanied the trial: the first one to monitor the setting in case of emergency and to measure time, and the other to record the mechanical monitoring instrument showing the effectiveness of resuscitations.

### 2.3. Randomisation

The method order was randomized with a coin toss prior to each dive.

(1) **Schmitz–Hinkelbein method**

In the first method (Schmitz–Hinkelbein method, SHM), the patient is in a supine position on the performer’s knees for stabilization, similar to the CPR method performed on Earth. It is important that the rescuer flexes his/her hips properly to provide a stable base under the patient. Chest compressions are conducted using both hands according to CPR guidelines for normogravity (Figure 1).

(2) **Cologne method**

The second method (Cologne method, CM) is similar to the first, but chest compressions are conducted with the elbow. The free arm of the rescuer can be used to stabilize the patient (Figure 2).

### 2.4. Data Collection

Participants were asked to perform external chest compressions for at least 60 s for each method. The additional diver provided a hand signal to the operator to begin compressions and also timed the attempts. The sequence to perform each method was determined by an additional diver by flipping a coin prior to the dive and signaling the result by a hand signal while diving.

### 2.5. Video Analysis

As a consequence of the submerged model, technical evaluation of CPR parameters by software was not possible. Video clips of at least 60 s per method were recorded (using the manual compression screen on the manikin) with a GoPro^®^ HERO4 (GoPro Inc., San Mateo, CA, USA) in a water-resistant case. All investigators were experienced in the performance of CPR and had undertaken preliminary training on a manikin during ground-based training/trials. In total, 30 videoclips were recorded (15 for each method). Recorded videoclips were screened and analyzed by two experienced emergency physicians independently.

### 2.6. The Primary Endpoint Was as Follows:

- Compression rate (defined as compression of the thorax) (per min).

### 2.7. The Secondary Endpoints Were as Follows:

- Number of chest compressions (*n*);
- Correct depth (defined as min. 50 to max. 60 mm of depth) (mm);
- Number of periods with no chest compression above 2 s (no-flow time) (*n*);
- Correct thoracic release between compressions (high = more than 66%; moderate = 33–65%; low = 0–32% of number of chest compressions with release of more than 4 cm according to indicator on manikin).

Data of primary and secondary endpoints were assessed and recorded independently. In cases of discrepancy, a third physician was consulted and majority counts were used.

### 2.8. Statistics

Case number determination (Cohen’s d > 0.8, alpha 0.05 and statistical power 0.8) revealed a required number of participants of fifteen for each method. For statistical analyses, data were processed with Excel for Mac 16.32 (Microsoft©, Redmond, WC, USA). Data were checked for normal distribution with the Kolmogoroff–Smirnov test, and differences were tested with an unpaired t-test. Results were considered significant if *p* < 0.05. All findings are presented as means ± standard deviations (*p*-value) if not stated otherwise.

### 2.9. Ethics and Registration

This study was registered on ClinicalTrials.gov (NCT04354883, 21 April 2020) and authorized by the ethical committee of the University Hospital of Cologne (19-1069_1, date: 1 April 2019).

## 3. Results

### 3.1. Subjects

Demographic parameters for female (*n* = 5) and male (*n* = 10) paramedics differed significantly for weight (63.3 ± 6.5 kg (mean BMI: 21.7) vs. 84.5 ± 14.1 kg (mean BMI: 26.1); *p* < 0.001) and age (22 ± 2 years vs. 32 ± 9 years; *p* < 0.001) but not for height (female, 170.7 ± 6.7 cm vs. male, 180.1 ± 8.9 cm; *p* < 0.001). All subjects held a valid diving license (OWD: 25%, CMAS *: 15%, CMAS **: 10%, and other: 50%).

### 3.2. Compression Rate

Fifteen participants conducted the Schmitz–Hinkelbein method (SHM). The average compression rate was 111.1 ± 6.3/min. The correct compression rate, defined as 100–120 compressions min^−1^, was achieved 90 ± 11% of the time.

Fifteen participants conducted the Cologne method (CM) with an average compression rate of 102 ± 8.3/min chest compressions per minute, and the expected compression rate was achieved 72 ± 23% of the time.

### 3.3. Chest Compression Depth

The expected 50–60 mm of chest compression depth was achieved 65 ± 23% of the time and 28 ± 27% of the time when performing SHM and CM, respectively.

### 3.4. Period of No Chest Compression >2 s (No-Flow Time)

Among the fifteen providers, a total of 6 and 4 periods of no-flow time were recorded, respectively.

### 3.5. Correct Thoracic Release between Compressions

For SHM, ten participants (66.6%) achieved a high rate of correct thoracic release, three participants (20%) showed a moderate rate of correct thoracic release, and two participants (13.3%) showed a low rate of correct thoracic release.

In performing CM, three participants (20%) showed a high rate of correct thoracic release, one participant (6.7%) showed a moderate rate of correct thoracic release, and eleven participants (73.7%) showed a low rate of correct thoracic release.

## 4. Discussion

The Schmitz–Hinkelbein method showed overall superior results for compression rate and compression depth associated with low rates of no-flow time and high rates of correct thoracic release in comparison to the second new method (CM).

### 4.1. General Considerations

The average compression rate for SHM was 111.1 ± 6.3 with a correct compression rate achieved 90 ± 11% of the time and fulfilled latest criteria for CPR-compression rate on Earth (100–120 compressions per minute) [14]. The average compression rate of the second new method was 102 ± 8 with a correct compression rate of 72 ± 23%, which was quite low and did not reach criteria for CPR in normogravity [14].

In recent studies, the Handstand method (HS) proved to deliver the most effective chest compressions with regards to the 2021 ERC guidelines [14]. The recently published international guidelines for CPR in microgravity [15] recommend the ER method as the primary method for basic life support in microgravity because of its advantages in feasibility and independence of cabin diameter. Normally, emergency equipment is stored near the Crew Medical Restraint System; thus, transport of a patient undergoing CPR is possible.

Moreover, our two new methods for CPR can be applied as first-approach methods, enabling transport of the patient to the Crew Medical Restraint System (CMRS).

As soon as the patient has been restrained on the CMRS, HS should be applied if not limited by the dimensions of the spacecraft and provider height because it yields the best-quality manual chest compressions in microgravity thus far [17].

The feasibility of a CPR method (in space) is a fundamental component of the health system (crew). Although recent data show that, throughout Europe, there are important differences in Emergency Medical Service systems [18], it is well established that an early start on chest compressions (as soon as possible) is vitally important after cardiac arrest and is correlated with a higher probability of survival [10,19]. As a first approach, HS, ER, and RBH are independent of any resources (and initially superior to those methods that require the patient to be restrained). Our new methods also do not require equipment. After starting CPR in space, the patient should be restrained as soon as possible because of its substantial benefits. Taking into account the transport of the patient to locations of both medical equipment and the CMRS, as well as the time required to restrain the patient, SHM could theoretically produce the best outcome [20].

Most current methods require equipment or methods of securing the patient and/or rescuer in order to begin CPR in the event of cardiac arrests. These methods need time to be implemented and will not be feasible in all future space vehicles because of differences in spacecraft diameter and availability of equipment. Methods for CPR have to be universally usable independent of room size or available equipment (e.g., patient restraining system), or should at least be able to guarantee high-quality CPR until needed equipment is retrieved.

The advantages of the two new methods are mainly the usability for initial CPR as single-helper or two-helper methods, and as reliable methods for ensuring high-quality chest compressions that could increase the probability of surviving cardiac arrest [16].

A major limitation is always a lack of post-resuscitation care after cardiac arrest in space. A recent study showed data of an automatic external chest compression device (ACCD) evaluated during a parabolic flight in 2021. Although transportation costs will be extremely high for ACCD, the use of an ACCD allows continuous delivery of high-quality CC in microgravity and hypergravity conditions [17].

### 4.2. Number of Chest Compressions and Compression Rate

In order to maintain adequate cardiac output, the compression rate (CR) is crucial [17]. In comparison, the HS method achieved the highest average rate (115.4 ± 12.1/min). Almost every performed method met the minimum requirement in terms of compression rate: ER (104.6 ± 6.0/min), STD (100.0 ± 3.0/min), and SM (102.6 ± 12.1/min) [21]. Only the RBH method did not meet the required criteria (94.7 ± 5.4/min) according to universal CPR guidelines [18].

Our first evaluated method achieved an average rate (100.5 ± 14.4/min) and our second method did not meet the required criteria (73.9 ± 25.5/min). Thus, compression rates showed significant differences (*p* < 0.001) between these two methods in our study, and only SHM met required criteria according to universal CPR guidelines [18].

### 4.3. Compression Depth

Prior data showed that the HS method was, in terms of compression depth, superior (44.9 ± 3.3 mm) [22]. Furthermore, the RBH (39.8 ± 6.3 mm) [22] and ER methods (35.6 ± 6.7 mm) [18] showed good results. Similarly to CPR in normogravity, with the operator kneeling next to the patient, both conventional methods STD (19.8 ± 11.2 mm) and SM (30.7 ± 11.9 mm) showed insufficient chest compressions. Table 1 summarizes the mathematical estimation parameters of known CPR methods with the new methods evaluated in our study.

Our two evaluated methods, both with a rescuer in a kneeling position next to the patient, showed improved depth rates. The first method (SHM) showed that almost two of three chest compressions (65 ± 23%; median 70%) had correct depths (50–60 mm). The second method showed worse depth rates with only one of four chest compressions (28.0 ± 2.7%; median 22%) with correct depth rates. The depth of chest compressions showed significant differences (*p* < 0.001) between these two methods in our study.

### 4.4. Limitations

Although compression rates primarily depend on which CPR method is used, compression depth depends not only on the method but also on the manikin used, the performers’ demographics, and the method of simulated microgravity. Although data for gender differences were found to be not significant [23], different types of manikins with variable resistance may affect results in compression depth and correct thoracic release. To what extent the differently used manikin types could influence the quality of CPR is still unknown.

The resistance of water might have also influenced compression rates, because the second method was the only method that did not meet criteria for correct compression rate. Moreover, the impact of environment on physical strain may complicate comparability of different CPR methods for resuscitation in space, as recent data showed a significant reduction in quality of resuscitation during an alpine rescue mission scenario at high altitudes due to physical strength [24].

The ERC recommends a cycle of 2 min of continuous chest compressions [14]. As parabolic flight can only enable a cycle of up to 22 s, we found that a cycle of one minute can conclude data of endpoints as, i.e., criteria of chest compressions is counted on a per-minute basis. Exhaustion may increase with longer cycles of chest compressions.

There are different methods to simulate microgravity on earth. Some studies used a body suspension device (BSD) [21], which was developed by the John Ernsting Aerospace Physiology Laboratory, Microgravity Centre, Pontifical Catholic University of Rio Grande do Sul. In contrast to parabolic flights [22] with limited study time (max. 22 s per parabola), longer periods of continuous chest compressions are possible with a BSD. Due to the fact that some studies used parabolic flights to simulate microgravity, data for prolonged CPR can only be compared conditionally [25]. Moreover, there are no data concerning the exhaustion of the operator after more than 3 min of CPR in a microgravity environment.

Moreover, the height of the performers seems to influence the quality of chest compressions, as one recent study showed in a normogravity setting [26].

The relative success of the first CPR method (SHM) examined in this study suggests that it may be more appropriate than procedures currently known for CPR in space. Therefore, further empirical examination, such as evaluation of the first and second methods with electronic data collection during parabolic flights, should be of future interest.

## 5. Conclusions

Thus far, five different methods for CPR in microgravity have been described [17]. Regarding compression depth, no method achieved the requirements of the current guidelines [14]. The Schmitz–Hinkelbein method showed sufficient compression rate and depth and seemed promising, but it needs evaluation in an authentic microgravity setting, such as during a parabolic flight. In order to provide high-quality CPR in space, a combination of different methods can be applied. The first new method (SHM) evaluated in this study seems to have some advantages and can be applied as a first-approach method since chest compressions can be conducted immediately without any equipment. Moreover, the performance of this method can be practiced prior to space missions and is similar to performing CPR on Earth.

## Figures and Tables

**Table 1:** Number of chest compression and depth rates of known methods [16] and evaluated methods in this study.

Method	Number of Chest Compressions (/min)	Correct Compression Depth (50–60 mm)
***New Methods***		
*Schmitz–Hinkelbein method*	*100.5 ± 14.4*	*0.65 ± 0.23*
*Cologne method*	*73.9 ± 25.5*	*0.28 ± 0.27*
***Existing Methods***		
Handstand method	115.4 ± 12.1	0.91 ± 0.07
Evetts–Russomano method	104.6 ± 5.4	0.74 ± 0.1
Reverse bear hug method	94.7 ± 5.4	0.82 ± 0.13
Side straddle method	100.0 ± 3.0	0.50 ± 0.28

## Data Availability

The data presented in this study are available on request from the corresponding author. The data are not publicly available due to privacy issues.

## Abbreviations

BSD	Body Suspension Device
CMAS	Confederation Mondiale des Activites Subaquatiques
CM	Cologne method
CMO	Crew Medical Officer
CMRS	Crew Medical Restraint System
CPR	Cardiopulmonary Resuscitation
CD	Compression Depth
CR	Compression Rate
ER	Evetts–Russomano method
ESA	European Space Agency
ETI	Endotracheal Intubation
GA	General anesthesia
HS	Handstand method
ISS	International Space Station
LEO	Low-earth Orbit
NASA	National Aeronautics and Space Administration
PADI	Professional Association of Diving Instructors
OWD	Open Water Diver
RBH	Reverse bear hug method
RCT	Randomized Controlled Trial
SHM	Schmitz–Hinkelbein method
SSI	Scuba Schools International
